# Revisiting policy on chronic HCV treatment under the Thai Universal Health Coverage: An economic evaluation and budget impact analysis

**DOI:** 10.1371/journal.pone.0193112

**Published:** 2018-02-21

**Authors:** Waranya Rattanavipapong, Thunyarat Anothaisintawee, Yot Teerawattananon

**Affiliations:** 1 Health Intervention and Technology Assessment Program (HITAP), Department of Health, Ministry of Public Health, Nonthaburi, Thailand; 2 Department of Family Medicine, Faculty of Medicine, Ramathibodi Hospital, Bangkok, Thailand; Kaohsiung Medical University, TAIWAN

## Abstract

Thailand is encountering challenges to introduce the high-cost sofosbuvir for chronic hepatitis C treatment as part of the Universal Health Care’s benefit package. This study was conducted in respond to policy demand from the Thai government to assess the value for money and budget impact of introducing sofosbuvir-based regimens in the tax-based health insurance scheme. The Markov model was constructed to assess costs and benefits of the four treatment options that include: (i) current practice–peginterferon alfa (PEG) and ribavirin (RBV) for 24 weeks in genotype 3 and 48 weeks for other genotypes; (ii) Sofosbuvir plus peginterferon alfa and ribavirin (SOF+PEG-RBV) for 12 weeks; (iii) Sofosbuvir and daclatasvir (SOF+DCV) for 12 weeks; (iv) Sofosbuvir and ledipasvir (SOF+LDV) for 12 weeks for non-3 genotypes and SOF+PEG-RBV for 12 weeks for genotype 3 infection. Given that policy options (ii) and (iii) are for pan-genotypic infection, the cost of genotype testing was applied only for policy options (i) and (iv). Results reveal that all sofosbuvir-based regimens had greater quality adjusted life years (QALY) gains compared with the current treatment, therefore associated with lower lifetime costs and more favourable health outcomes. Additionally, among the three regimens of sofosbuvir, SOF+PEG-RBV for genotype 3 and SOF+LDV for non-3 genotype are the most cost-effective treatment option with the threshold of 160,000 THB per QALY gained. The results of this study had been used in policy discussion which resulted in the recent inclusion of SOF+PEG-RBV for genotype 3 and SOF+LDV for non-3 genotype in the Thailand’s benefit package.

## Introduction

Around 150 million people worldwide have hepatitis C viral (HCV) infection with most cases in East and Central Asia [1]. Thailand, a high-middle income country in South East Asia, is one of the countries with the highest number of HCV patients, with approximately 759, 000 cases [2]. The prevalence of HCV infection is 2.8% which is increased to 8.4% in patients with Human Immunodeficiency Virus (HIV) infection in adults aged 21–60 years. In addition, Thailand is also found to have a unique HCV genotype 3 prevalence, although HCV genotype 1 is most common globally [3].

Chronic HCV infection can cause liver inflammation and tissue scarring which can lead to liver cirrhosis and hepatocellular carcinoma if untreated [4]. For HCV treatment, goal is to reduce mortality and morbidity from end-stage liver disease and hepatocellular carcinoma by achieving the virological cure, defined as sustained virological response (SVR).

Last 2011, Thai National List of Essential Medicines (NLEM), the pharmaceutical reimbursement list for the Universal Healthcare Coverage (UHC) scheme, introduced peginterferon alfa in combination with ribavirin for the treatment of chronic HCV infection. This was the first national policy supporting the treatment of chronic hepatitis C in Thailand which prompted an increase in access of Thai chronic HCV patients to the free treatment, although majority of the patients still remain undiagnosed.

Sofosbuvir, an oral nucleotide polymerase inhibitor, was approved by the US Food and Drug Administration (FDA) in December 2013 and subsequently by Thai FDA in 2015. American Association for the Study of Liver Diseases (AASLD) and World Health Organization (WHO) guidelines currently recommend Sofosbuvir-based regimens as the standard treatment of chronic HCV infection. The recommendation is found to have high efficacy rates in terms of increased SVR, shorter treatment duration and lower rate of adverse drug reaction compared to peginterferon alfa in combination with rivabirin [5].

The high cost of sofosbuvir is a major barrier in accessing the medicine in both resource-rich and resource-poor settings [6]. A patent-holder of sofosbuvir has entered licensing deals with several manufacturers in India who are developing generic versions of the drug, but the deals exclude majority of the middle- income countries, including Thailand [7].

In 2015, an appeal for reevaluation of the treatment policy for HCV was raised by physicians, civil society and patient groups to the Thai government. Consequently, Health Intervention and Technology Assessment Program (HITAP) was requested by the Subcommittee for Development of NLEM to assess the value for money and potential budget impact of sofosbuvir-based regimens compared to the currently included treatment under the Thai UHC such as peginterferon-based regimens.

Given the unique HCV infection genotype prevalence in Thailand, policy makers are particularly interested to assess whether sofosbuvir-based regimens signifies a better value for money for pan-genotypic treatment regimen compared to genotype-specific treatments, as the former could save cost of HCV genotype testing. Results of this study can benefit other low and middle income countries (LMICs) facing similar challenges in prioritising care for chronic hepatitis C patients.

## Materials and methods

### Analyses and model overview

A model-based economic evaluation approach was used to estimate all costs and health outcomes for treatment-naïve adult patients with chronic HCV infection. Health outcomes were expressed in terms of quality adjusted life years (QALYs). The structure of the model was based from the previous model of hepatitis C treatment recommendation in Thailand [8] which was modified to include anemia–the most common adverse event in treating chronic hepatitis C.

The Markov state-transition model used considered a hypothetical cohort of patients who could remain in the same state or move continuously across health states from cycle to cycle based on transitional probabilities until death. The lifetime time horizon with a yearly cycle length to advance time was applied.

The model simulated progression of chronic HCV infection to compensated cirrhosis, decompensated cirrhosis, hepatocellular carcinoma and death. Starting with non-cirrhotic patients who received antiviral treatment, transition was based on response to HCV treatment: patients who achieved SVR at (1) 24 weeks after peginterferon-based therapy or (2) 12 weeks after receiving sofosbuvir-based therapy. Patients who did not achieve SVR transitioned into the natural history phases of HCV infection.

The treatment for chronic HCV infection comprised of four regimens: (i) current practice–peginterferon alfa (PEG) and ribavirin (RBV) for 24 weeks in HCV genotype 3 and 48 weeks for other genotypes; (ii) Sofosbuvir plus peginterferon alfa and ribavirin (SOF+PEG-RBV) for 12 weeks; (iii) Sofosbuvir and daclatasvir (SOF+DCV) for 12 weeks; (iv) Sofosbuvir and ledipasvir (SOF+LDV) for 12 weeks for patients with chronic HCV non-3 genotypes and SOF+PEG-RBV for 12 weeks for chronic HCV genotype 3 infection. The fourth regimen was added to increase available evidence on the efficacy of sofosbuvir and ledipasvir for treating chronic HCV genotype 3 infection [9].

Given that policy options (ii) and (iii) are for pan-genotypic infection, the cost of genotype testing was applied only for policy options (i) and (iv). Furthermore, the outcomes of interest are lifetime costs, QALYs gained, and the incremental cost-effectiveness ratio (ICER) in Thai Baht (THB) per QALY gained.

Societal perspective was considered and 3% discount rate was applied for estimating the future costs and benefits, as recommended in the health technology assessment guidelines of Thailand [10].

### Model parameters

#### Health state transitional probabilities

Transitional probabilities between health states were obtained from previously published economic evaluation [8]. To acquire probabilities of treatment-associated anemia, data from previous studies were reviewed through meta-analysis using STATA version 14. The details of meta-analysis is found in the attached S1 –authors’ previous publication [11]. Additionally, risk of anemia for each of the regimen was calculated (Table 1).

**Table 1:** Probabilities of treatment-associated anemia.

Probabilities	Mean	SE	Distribution*
Anemia associated with PEG-RBV	0.25	0.05	Beta
Anemia associated with SOF+PEG-RBV	0.18	0.03	Beta
Anemia associated with SOF+DCV	0	0	Beta
Anemia associated with SOF+LDV	0.009	0.005	Beta

*Probability distributions for employing uncertainty analysis in economic evaluationSE—Standard error; PEG—Peginterferon alfa; RBV—Ribavirin; SOF—Sofosbuvir; DCV—Daclatasvir; LDV—Ledipasvir

### Treatment efficacy

Data from previous systematic review was reanalysed [11]. Efficacy of drug combination in all HCV genotypes was reviewed and reported as SVR rates. However, the study selection focused on genotype 3 and non-3 genotypes (genotypes 1 and 6)–the most prevalent type of HCV in Thailand [9]. Twelve studies identified by clinical experts were included in the study. This resulted in the inclusion of 23 randomized controlled trials which compared efficacy of treatment regimens of interest in treatment-naïve chronic HCV adult patients with genotype 3 or non-3 genotypes. Further, probability of achieving SVR for each treatment regimen was pooled using the STATA program (Table 2). The details of revised selection criteria and flow chart of study selection are found in the attached S1 Text.

**Table 2:** Efficacy of antiviral combination therapy for treatment of chronic HCV infection.

Regimen	Sustained Virological Response* (%)			
	HCV genotype 3 (95% CI)	Reference	HCV genotype 1&6 (95% CI)	Reference
PEG-RBV	83 (77–89)	[12–15]	68 (60–76)	[12–22]
SOF + PEG-RBV	95 (90–98)	[23]	92 (89–94)	[24–27]
SOF + DCV	96 (91–99)	[28]	100 (94–100)	[29]
SOF + LDV	64 (42–82)	[30]	95 (93–98)	[27, 31–33]

*Sustained virological response (SVR) refers to SVR at 24 weeks post-treatment of peginterferon alfa and ribavirin or at 12 weeks post-treatment of sofosbuvir-based regimens

### Costs

The societal viewpoint was adopted in the calculation of the costs. Direct medical and direct non-medical costs incurred from each treatment regimen were taken into account. Direct medical costs covered cost of treating chronic HCV infection, anemia, HCV complications, laboratory tests for investigation and monitoring including HCV genotyping (for peginterferon-based regimen and sofosbuvir with ledipasvir), and outpatient fees. Direct non-medical costs included travel and food costs for patients and caregivers, personal facility costs, and opportunity costs incurred by patients.

The cost of antiviral agents was based on the proposed price of pharmaceutical companies to the Subcommittee for Development of NLEM. Other data on costs were collected from literature review and national databases. All costs were converted to 2016 values based on the time of data analysis using the Thai consumer price index [34] and presented in Thai Baht (THB) (approximately THB 36 = USD 1 in 2016). For international comparison, costs were converted into international dollars using the purchasing power parity (PPP) conversion rate, where a PPP 2016 dollar is worth 12.146 THB [35]. Costs used in the model are shown in Table 3.

**Table 3:** Cost parameters*.

Costs	Mean (THB)^†^	Distribution^§^	Reference
**Antiviral combination therapy (per course)**			
PEG-RBV for 24 weeks	75,600	Gamma	Drug companies
PEG-RBV for 48 weeks	151,200	Gamma	Drug companies
SOF + PEG-RBV for 12 weeks	163,800	Gamma	Drug companies
SOF + DCV for 12 weeks	252,000	Gamma	Drug companies
SOF + LDV for 12 weeks	166,500	Gamma	Drug company
**Laboratory tests for investigation and monitoring (per course)**			
HCV genotype testing	5,410	Gamma	[36]
PEG-RBV for 24 weeks	17,100	Gamma	[36, 37]
PEG-RBV for 48 weeks	20,200	Gamma	[36, 37]
SOF + PEG-RBV for 12 weeks	8,700	Gamma	[36, 37]
SOF + DCV for 12 weeks	8,700	Gamma	[36, 37]
SOF + LDV for 12 weeks	14,100	Gamma	[36, 37]
**Treatment costs of HCV complication (per year)**			
Chronic HCV infection	72,000	Gamma	[38]
Compensated cirrhosis	80,000	Gamma	[38]
Decompensated cirrhosis	148,300	Gamma	[38]
Hepatocellular carcinoma	183,800	Gamma	[38]
Anemia	8,317	Gamma	[36, 39]
**Direct-non medical costs**			
Chronic HCV infection (per year)	4,470	Gamma	[36]
Compensated cirrhosis (per year)	4,380	Gamma	[36]
Decompensated cirrhosis (per year)	6,060	Gamma	[36]
Hepatocellular carcinoma (per year)	9,900	Gamma	[36]
Hospital visit, outpatient service (per visit)	670	Gamma	[36]

*The number is rounded up.

^†^Sum of the aggregate amount of costs; standard errors of unit costs are not reported.

^§^ Probability distribution for employing uncertainty analysis in economic evaluation

THB—Thai Baht

### Health-related quality of life

Utility values of patients with chronic HCV infection and complications were gathered from a systematic review and meta-analysis of utility values [40] in Table 4. QALY is estimated as health outcome by multiplying utility value (health-related quality of life) and life years.

**Table 4:** Utility parameters.

Health states	Mean	SE	Distribution*	Reference
Chronic HCV infection	0.73	0.0011	Beta	[40]
Compensated cirrhosis	0.70	0.0020	Beta	[40]
Decompensated cirrhosis	0.58	0.0020	Beta	[40]
Hepatocellular carcinoma	0.58	0.0023	Beta	[40]
Anemia in chronic HCV infection^†^	0.61	0.0018	Beta	[40, 41]

*Probability distribution for employing uncertainty analysis in economic evaluation

^†^Analysis of secondary data collected by the authors. Subtraction of disutility due to anemia from utility value of chronic HCV infection

### Uncertainty analyses

Uncertainty analyses were employed to determine whether the results are robust to the differences that arise from parameter uncertainty. In one-way sensitivity analysis, an individual parameter was varied between a low and high value, and all other parameters were held constant. Probabilistic sensitivity analysis (PSA) using Bayesian framework involved the sampling of all model parameters from its distribution. The PSA simulation was run 1,000 times to generate result. Uncertainty about the parameters of the model was incorporated in the estimates of expected cost and QALYs which was presented in cost-effectiveness acceptability curve (CEAC) and tornado chart.

### Budget impact analysis

The annual direct medical cost was based on Markov model. Moreover, the secondary data sources was used for estimating the budget impact which include prevalence of HCV infection in Thai population, proportion of patients with HCV infection genotype 3 and non-3 genotype [42], and proportion of patients who are eligible to medical conditions (METAVIR fibrosis score ≥ 2) for chronic HCV treatment under NLEM [37, 43]. Budget impact analysis comparing different treatment strategies was calculated based on the following scenarios:

- Adult patients with chronic HCV infection
- HCV infection prevalence, average for all age groups is 0.39%
- Proportion of patients with HCV infection genotype 3 and non 3 genotype is 48.12% and 51.88% respectively
- Proportion of patients who had METAVIR fibrosis score ≥ 2 accounts for 43.2% [43]
- Five percent treatment coverage which is recommended from expert panel as similar to current situation regarding access to standard treatment of chronic HCV infection [44]
- Payer perspective
- Time horizon of 10 years
- No discounting of costs
- A closed cohort budget impact model

## Results

### Cost-utility analysis

**Fig 1:**
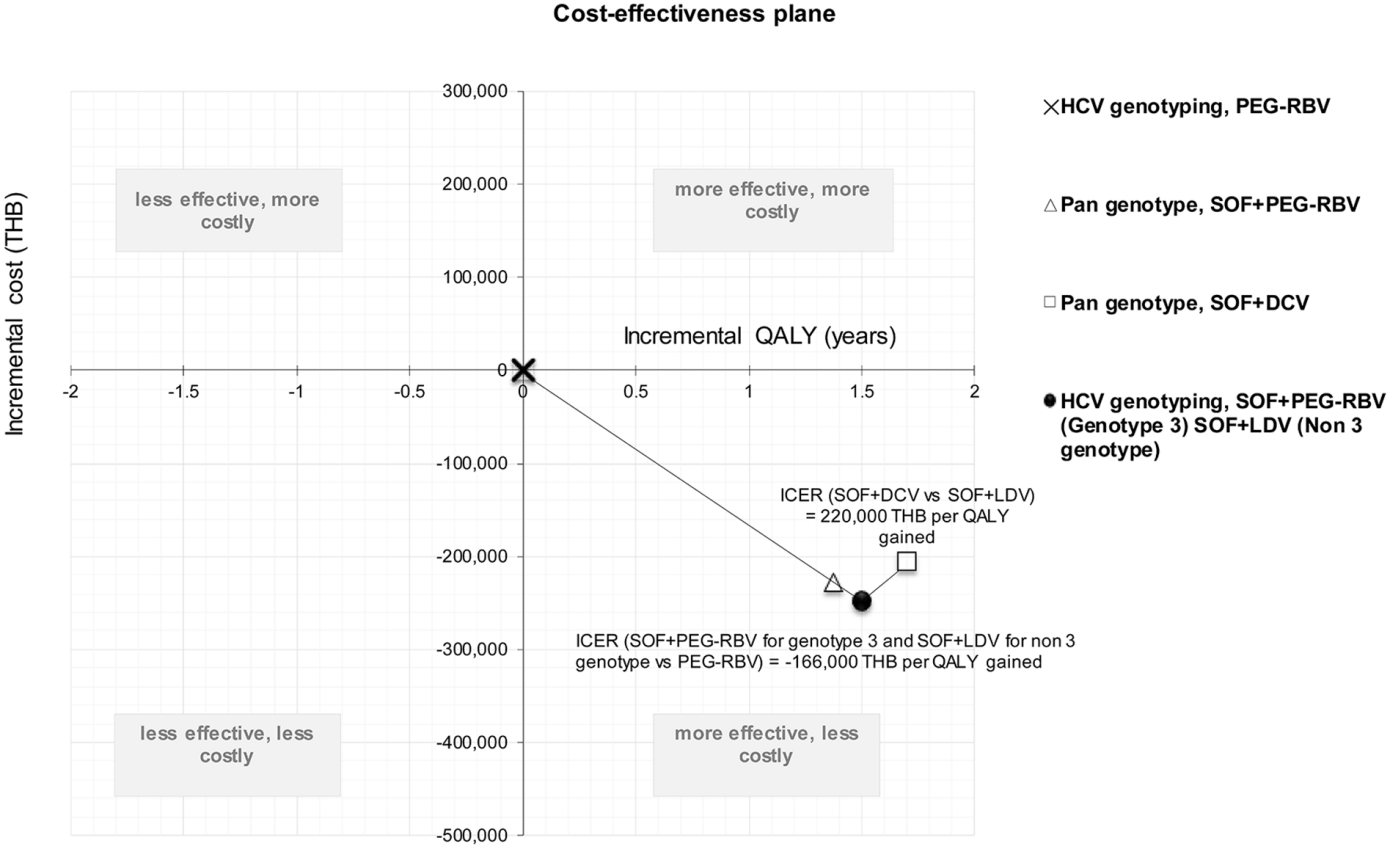
Cost-effectiveness plane of lifetime cost and effectiveness of four treatment strategies.

The model simulates lifetime of chronic HCV infection patients and was classified by HCV genotypes: genotype 3 and non-3 genotype. The cumulative costs, QALYs, and ICER were calculated based on a weight-based proportion of HCV genotypes (Fig 1).

For base-case analysis, SOF+PEG-RBV regiment for HCV infection genotype 3 and SOF+LDV for non-3 genotype had the lowest lifetime cost of 248,000 THB. SOF+DCV combination therapy for all genotypes was found to be the most efficacious at 21.36 QALYs.

In SOF+PEG-RBV, SOF+DCV, PEG-RBV regimen, lifetime cost was 271,000, 292,000, and 498,000 THB, respectively. QALYs generated from the PEG-RBV, SOF+PEG-RBV, SOF+ PEG-RBV (for HCV genotype 3) and SOF+LDV (for HCV non 3 genotype), and SOF+DCV was 19.66, 21.03, 21.16, and 21.36 years.

PEG-RBV served as the reference treatment at fixed point (0,0) (Fig 1). Compared to the reference point, all sofosbuvir-based regimens had lower costs and greater QALYs. It can be concluded that the ICER of all sofosbuvir-based regimens were more cost-saving compared to PEG-RBV.

Among sofosbuvir-based regimens, SOF+PEG-RBV in patients with HCV genotype 3 and SOF+LDV in patients with HCV non-3 genotype had excellent ICERs (cost-saving intervention), which indicates to be the most cost-effective options based on the cost-effectiveness ceiling threshold of 160,000 THB per QALY gained, as recommended by the Subcommittee for Development of the NLEM [45].

It should be noted that compared to the SOF+PEG-RBV (HCV genotype 3) and SOF+LDV (HCV non-3 genotype), SOF+PEG-RBV had higher costs and lower QALYs; therefore, it was completely dominated. On the other hand, the SOF+DCV regimen had the ICER of 220,000 THB per QALY gained as it had higher health gain (QALY) but at a higher cost.

### Uncertainty analyses

**Fig 2:**
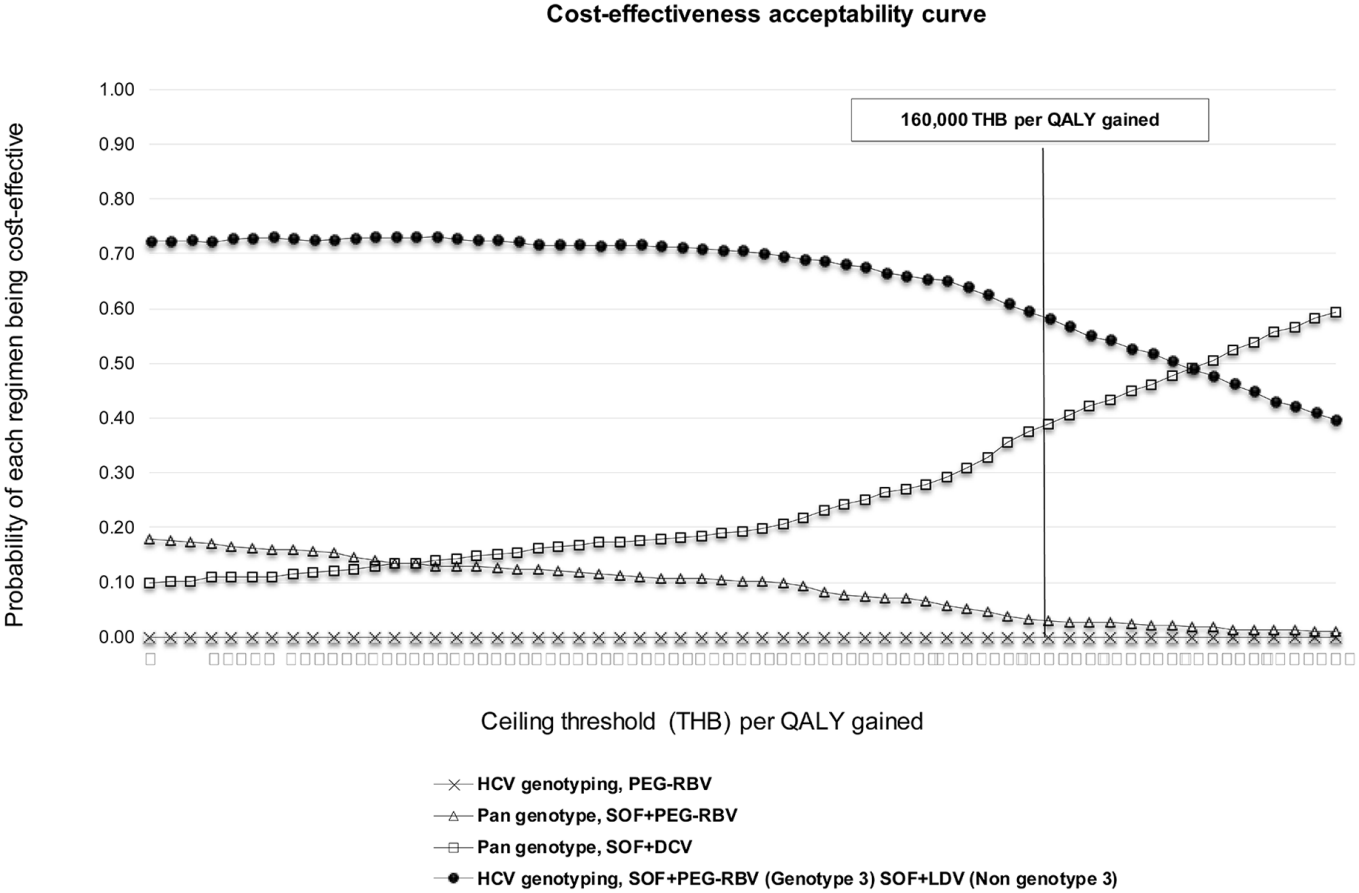
Acceptability curves of the cost-effectiveness at the different ceiling threshold of four treatment strategies for chronic HCV infection.

PSA result from the 1,000 simulations is presented as cost-effectiveness acceptability curves which show the relationship of the probability of each treatment being cost-effective versus ceiling threshold per additional one QALY (Fig 2). Result indicates that the regimen of SOF+PEG-RBV in patients with genotype 3 and SOF+LDV in patients with non-3 genotype was the most cost-effective treatment in 58% of the simulations. This was based on Thai ceiling threshold of 160,000 THB per QALY gained. In addition, it can be clearly seen in the figure that the cost-effectiveness of SOF+DCV regimen for all genotypes increases in correlation with the ceiling threshold.

**Fig 3:**
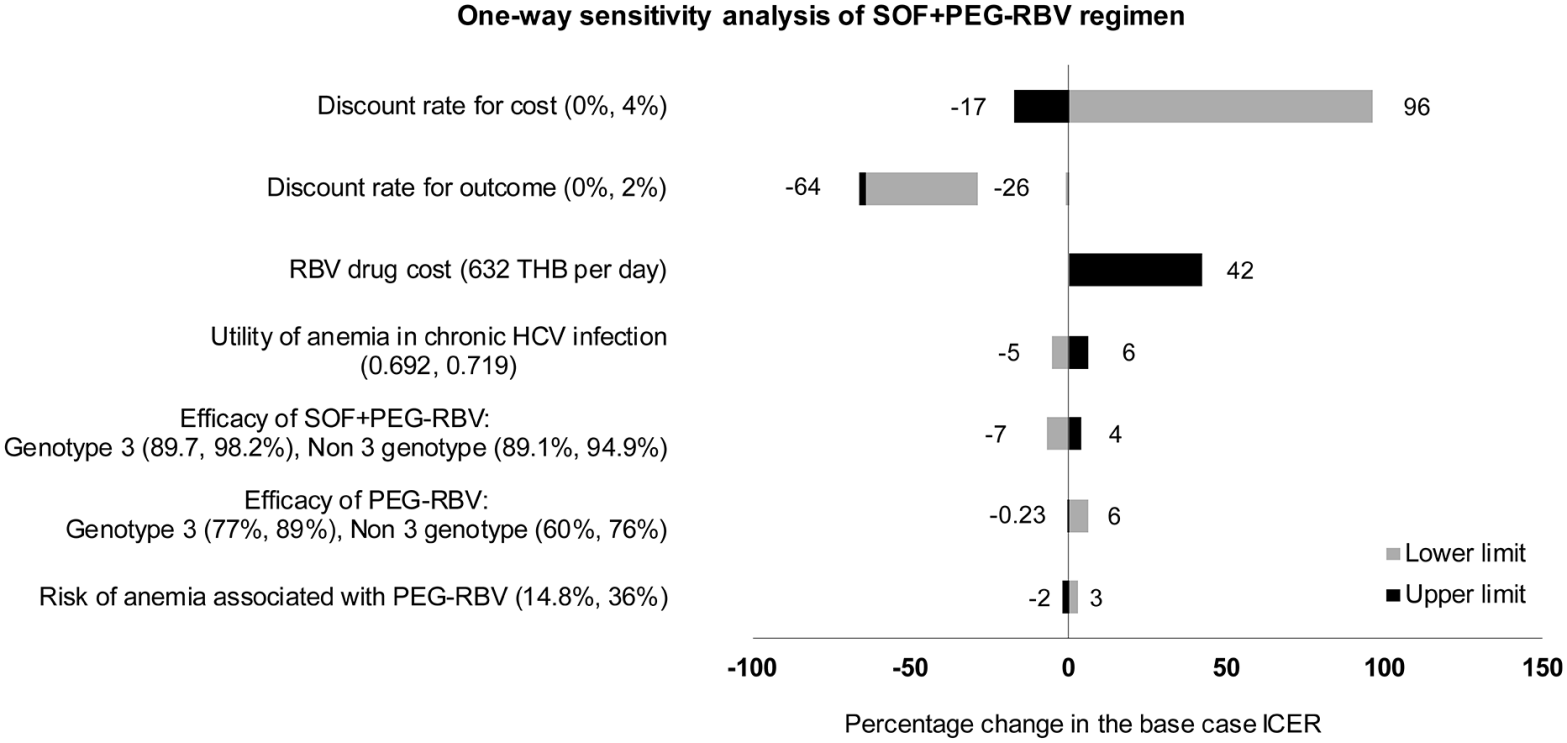
Results of one-way sensitivity analysis of SOF+PEG-RBV regimen compared to standard treatment (PEG-RBV regimen).

**Fig 4:**
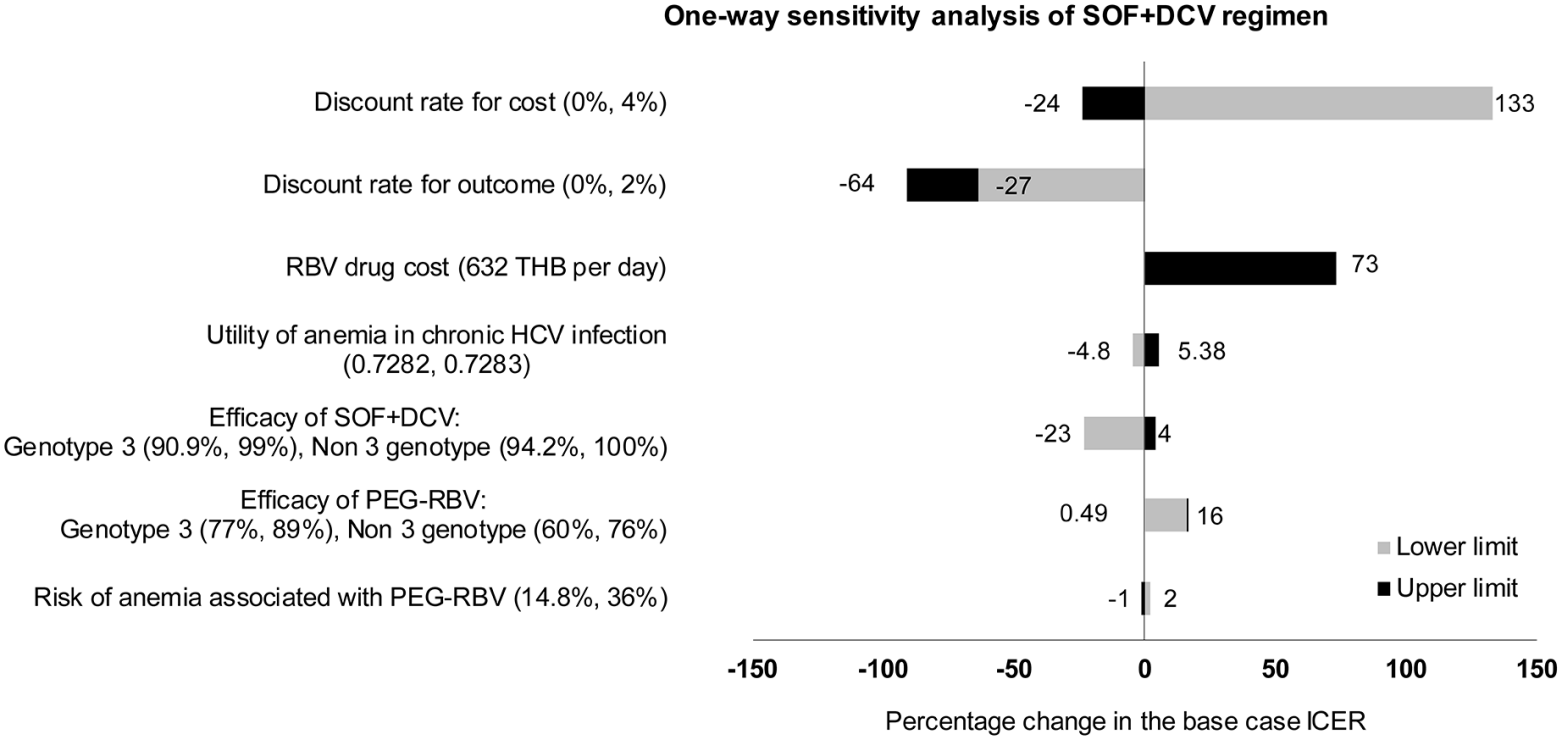
Results of one-way sensitivity analysis of SOF+DCV regimen compared to standard treatment (PEG-RBV regimen).

**Fig 5:**
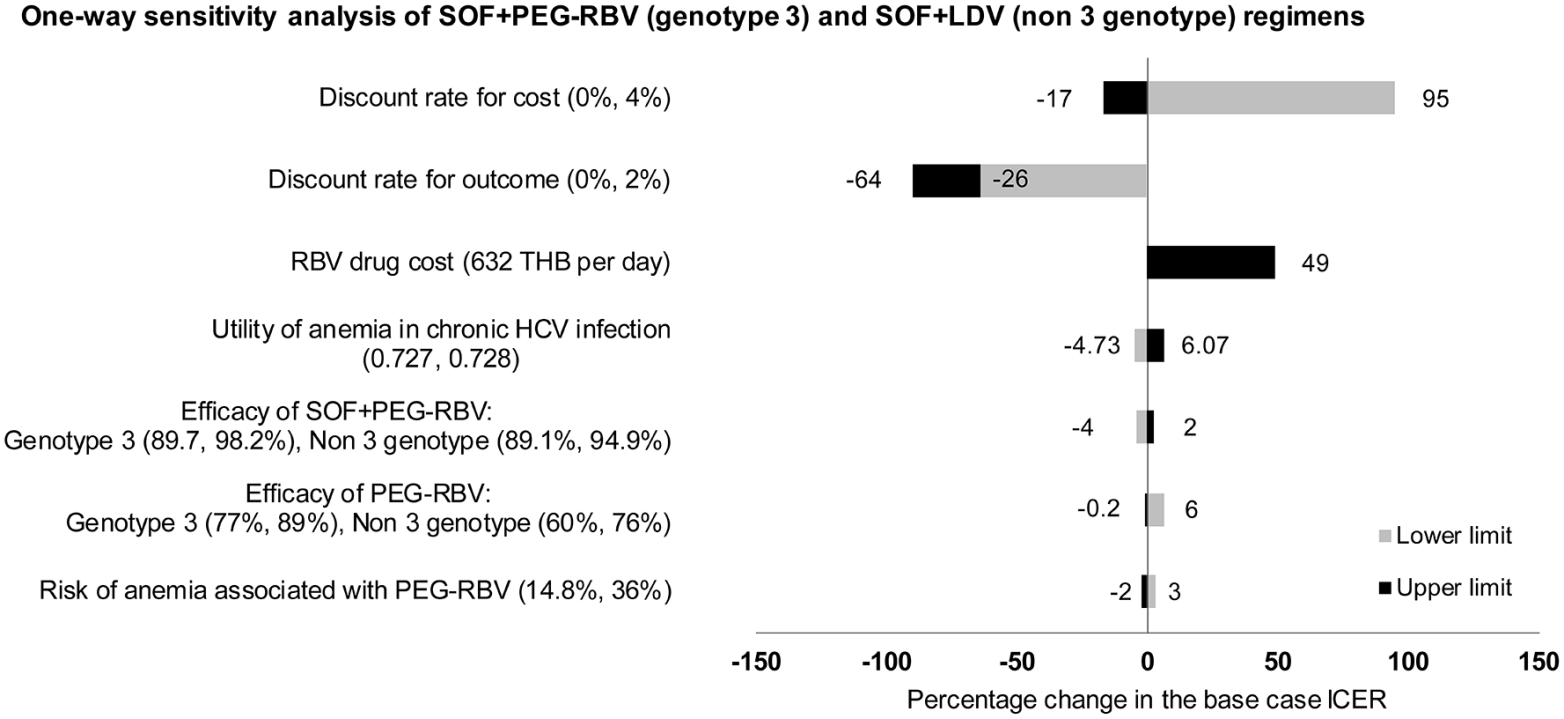
Results of one-way sensitivity analysis of SOF+PEG-RBV (genotype 3) and SOF+LDV (non-3 genotype) compared to standard treatment (PEG-RBV regimen).

For all three sofosbuvir-based regimens, one-way sensitivity analyses exhibited similar trend as shown in Figs 3, 4 and 5. The most influential parameter was found to be the discount rates for cost and outcome. Second is the cost of ribavirin which is currently provided free with peginterferon alfa. ICER of sofosbuvir-based regimens varies between 42%-73% from the base case analysis, with ribavirin cost varying from 632 THB per day. Other variables that had impact to the results include utility of anemia in chronic HCV infection, efficacy of drugs, and risk of anemia associated with PEG-RBV. None of the variations would affect the result that sofosbuvir-based regimens were more cost-saving options compared to PEG-RBV.

### Budget impact analysis

The population (aged 18–65 years) of Thailand is 44,225,116 as of 2015. The number of eligible chronic HCV patients who have access to HCV treatment is projected from this figure to estimate the budget impact to adopt the different alternative treatments for chronic HCV infection (Table 5).

**Table 5:** Expected number of chronic HCV patients used to estimate the budget impact.

	Patients with HCV genotype 3 (48.12%)	Patients with HCV non-3 genotype (51.88%)	Total patients
Patient with chronic HCV infection (prevalence = 0.39%)	82,995	89,482	172,478
Eligibility criterion, patient who had METAVIR fibrosis score ≥ 2 (43.2%)	35,854	38,656	74,510
5% coverage	1,793	1,933	3,726

The total cost for drugs and treatment for patients with chronic HCV infection by different regimens for the next 10 years was projected, assuming that 3,726 patients (2% of patients) are treated (Table 6). It was found that the total budget impact to adopt sofosbuvir-based regimens for chronic HCV infection was less than the current spending on the standard treatment with PEG-RBV, with the exception of SOF+DCV regimen.

**Table 6:** Budget impact of adoption of new therapies for chronic HCV infection in THB (millions).

Year	HCV genotyping, PEG-RBV (standard treatment)			SOF+PEG-RBV for all genotypes			SOF+DCV for all genotypes			HCV genotyping, SOF+PEG-RBV (Genotype 3) and SOF+LDV (Non 3 genotype)		
	Drugs	Laboratory tests	Treatment*	Drugs	Laboratory tests	Treatment*	Drugs	Laboratory tests	Treatment*	Drugs	Laboratory tests	Treatment*
1	378	71	0	610	32	0	939	32	0	615	43	0
2–10	0	0	543	0	0	142	0	0	42	0	0	104
Total	992			784			1,013			762		

*Treatment costs due to patients who failed to therapy and continued to progress to HCV complications

Although sofosbuvir-based regimens would cost additional budget in the first year, it would save cost by reducing laboratory tests due to a shorter follow-up time and lower the treatment costs due to HCV complications (more than 70%). Consequently, compared to PEG-RBV alone, payers would need additional 232 million THB for SOF+PEG-RBV, 237 million THB for SOF+PEG-RBV and SOF+LDV, and 561 million THB for SOF+DCV regimens to cover drug costs for eligible patients. In addition, it is estimated that palliative care for 95% of patients who cannot access to the treatment would cost approximately 107 billion THB over a 10-year period.

## Discussion and conclusions

This study demonstrates that the Thai government needs to change its current treatment policy for chronic HCV infection since all three new treatment policies using sofosbuvir-based regimens are likely to yield more health benefit with lower cost compared to the current policy.

Cost savings from HCV genotype testing do not outweigh clinical benefit from using genotype-specific treatment regimen as it shows that SOF+PEG-RBV for genotype 3 and SOF+LDV for non-3 genotype are the most cost-effective option in Thailand. Result reflects that SOF+DCV for all genotypes is the most clinical effective choice but a relatively higher drug cost leads to a lower cost-effectiveness value. However, allowing the health care purchaser to negotiate lower drug prices of SOF, DCV, and SOF+LDV should be an option. This is to ensure the finance sustainability. These findings support the recent revision of the Thailand Clinical Practice Guideline for Management of chronic hepatitis C which recommends sofosbuvir-based regimens for treating chronic HCV infection for all genotypes [46].

Notably, RBV cost caused uncertainty in choosing between option (iii) and (iv). If RBV cost is lowered, option (iv) would be more favourable, and vice versa. Meanwhile, HCV genotype testing cost makes no effect to this economic analysis which could be explain by the fact that the HCV genotype test is a one-off investment and its cost is only fraction of the treatment cost, accounting to 0.78–2.21% of the total lifetime treatment cost.

Similar to other settings, Thai decision makers are facing challenges in introducing sofosbuvir-based regimens for treating HCV infection. If the projection would be correct, treatment coverage of HCV infection would significantly increase to 10–30% of the total eligible population, which would lead to a huge financial burden to Thai UHC. Currently, the government is investing less than a billion THB on HCV. The budget would need to increase to 150% for 10% coverage, and 450% for 30% coverage for Thai patients to get better access to a more effective with lesser adverse events treatment options.

The financial burden could be even more challenged if the new policy would include treatment for patients previously treated with reinfection. Financial sustainability is a serious concern of the Subcommittee on Development of NLEM and at this point in time, the subcommittee is under price negotiation with companies on these medicines.

Results from this study are similar to other previous studies conducted mainly in high-income countries that demonstrate good value for money for sofosbuvir-based regimens compared to peginterferon-based choices [47, 48]. Additionally, this study informs that HCV genotype testing cost is not an important factor considering treatment of choice for chronic HCV infection. Based on comprehensive literature review, this is the first study exploring the effect of HCV genotype testing cost on cost-effectiveness results in LMICs.

This study poses some limitation on the methodology. First, it did not include the benefit of HCV treatment in preventing further transmission, as it would require a larger epidemiological data for disease dynamic model [49]. It is expected that the cost-effectiveness results would be more favourable if this public health benefit was taken account. Second, liver transplantation was not an option in the model because it is neither currently widely available in Thailand nor included the Thai UHC benefit package. Third, this study did not include the higher treatment costs if treatment of HCV reinfection from previously treatment patients was included. On the other hand, health benefit of this HCV treatment policy would be diminished if the treatment policy allows only once-in-lifetime treatment for chronic HCV infection. This means that re-infected patients could develop cirrhosis and liver cancer if they got new infection after effective treatment. In addition, the study of HCV treatment in HIV co-infected patients remains a topic of interest, which is a recommendation for further research. Lastly, proportion of patients who had METAVIR fibrosis score ≥ 2 accounts for 43.2% and was derived from a single study conducted in Thailand. Although this figure was verified by the Thai experts in the stakeholder consultation meetings held at the end of this study, it may be possible that this figure was overestimated as diagnosed patients with HCV infection. These patients are more likely to have more advanced liver disease than those whose infection has not be diagnosed. However, this parameter is unlikely to affect the cost-effectiveness results but may result in an overestimation of potential budget impact.

The results of this study had been widely used in policy discussion and price negotiation which resulted in the recent inclusion of SOF+PEG-RBV for genotype 3 and SOF+LDV for non-3 genotype in the Thai NLEM. Onwards 2018, Thai patients infected by HCV can have access to these two DAA regimens depending on HCV genotype test. Given that there are a number of middle-income countries unable to access to generic version of sofosbuvir, this study can provide insightful information for decision makers in those countries though there may be limited generalisability due to the current policy in each setting, drug and treatment costs and health preference. For example, Aggarwal et al. [7] demonstrates that offer HCV treatment with generic version of sofosbuvir in India represents a good value for money within 2 years, and ultimately becomes cost-saving within 10 years. Decision makers and technical advisors should be warranted when applying these findings to their settings.

## Supporting information

S1 Text Treatment efficacy. (DOCX)
