# Supplementary material for: Revisiting policy on chronic HCV treatment under the Thai Universal Health Coverage: An economic evaluation and budget impact analysis
Source: PLoS One. 2018 Feb 21;13(2):e0193112. doi: 10.1371/journal.pone.0193112 (PMC5821370; doi:10.1371/journal.pone.0193112)
Supplement: S1 Text — (DOCX) [file pone.0193112.s001.docx]

**Supporting Information**

**S1 text.** **Treatment efficacy and**

A systematic review and meta-analysis with the aim of comparing the treatment efficacy and hepatitis C treatment-induced anemia between different regimens for treating chronic HCV infection were conducted by authors and published elsewhere. This economic evaluation focused on the efficacy and adverse event in specific target population and of particular treatment regimens. Therefore, authors reviewed and reanalyzed the data from their own previous systematic review and meta-analysis study (1). Details are in the next section.

**Literature search**

The MEDLINE and Scopus databases up to 25 May 2015 was searched for randomized controlled trials of peginterferon alfa combined with ribavirin and three different sofosbuvir based regimens for chronic hepatitis C.

**Population of interest**

Treatment-naïve adults with chronic HCV genotype 3 and non-3 genotype (genotype 1 and 6)

**Treatment of regimens of interest**

This study focused on four different treatment regimens: (1) peginterferon alfa (PEG) and ribavirin (RBV), (2) sofosbuvir plus peginterferon alfa and ribavirin (SOF+PEG-RBV), (3) sofosbuvir and daclatasvir (SOF+DCV), and (4) sofosbuvir and ledipasvir (SOF+LDV).

**Study selection**

Two reviewers independently reviewed titles and abstracts for selecting the studies. In cases when a definite decision cannot be made based on the title and/or abstract alone, the full articles were retrieved for detailed assessment against the inclusion criteria. Disagreement between the two reviewers was decided by consensus with a third party.

**Selection criteria**

Individual study has to meet all of the specified inclusion criteria:

1. Randomized controlled trial
2. Studies published in English
3. Treatment-naive adult patients (aged 18 years and above) with HCV genotypes 1, 3 or 6
4. Comparing the efficacy of any pairs of treatment of regimens of interest
5. Measurement of outcome is sustained viral response (SVR) at weeks 12 for sofosbuvir-based regimens or weeks 24 for peginterferon-based regimen after the end of treatment

**Flow chart of study selection process**

**Outcomes**

The outcome of interest were

1. SVRs at weeks 12 (SVR12)
2. SVRs at weeks 24 (SVR24) and
3. Number of patients who develop anemia

**Statistical analysis**

A meta-analysis of outcomes of interest was performed using STATA version 14. Risk ratios (RR) of SVR12 and SVR24 were estimated. Incidence of anemia for each treatment regimen was pooled. Details of statistical analysis are described in previous publication (1).

**Reference**

Suwanthawornkul T, Anothaisintawee T, Sobhonslidsuk A, Thakkinstian A, Teerawattananon Y. Efficacy of second generation direct-acting antiviral agents for treatment naive hepatitis C genotype 1: a systematic review and network meta-analysis. PloS one. 2015 Dec 31;10(12):e0145953.
